# The Associations of Maternal and Neonatal Vitamin D with Dental Development in Childhood

**DOI:** 10.1093/cdn/nzy100

**Published:** 2019-03-07

**Authors:** Brunilda Dhamo, Kozeta Miliku, Trudy Voortman, Henning Tiemeier, Vincent WV Jaddoe, Eppo B Wolvius, Edwin M Ongkosuwito

**Affiliations:** 1 Department of Oral & Maxillofacial Surgery, Special Dental Care and Orthodontics; 2 The Generation R Study Group; 3 Departments of Epidemiology, Erasmus University Medical Centre, Rotterdam, the Netherlands; 4 Departments of Child Psychiatry, Erasmus University Medical Centre, Rotterdam, the Netherlands

**Keywords:** maternal vitamin D, neonatal vitamin D, dental maturation, mandibular teeth, dental age

## Abstract

**Background:**

Vitamin D influences the formation and mineralization of teeth.

**Objective:**

To investigate the association of maternal and neonatal vitamin D concentrations with the dental development of 10-y-old children, in a population-based prospective cohort study among 3,770 mothers and children in the Netherlands.

**Methods:**

Maternal venous blood samples were collected in the second trimester (median 20.4 weeks of gestation; range: 18.5–23.2 wk) whereas umbilical cord blood samples were collected immediately after delivery (median 40.1 weeks of gestation; range 35.9–42.3 wk). Dental development was defined using the Demirjian method. Multivariate regression models were built to analyze the studied associations.

**Results:**

High concentrations of 25-hydroxyvitamin D [25(OH)D] during midpregnancy (β: −0.04; 95% CI: −0.08, −0.01) and at birth (β: −0.06; 95% CI: −0.10, −0.02) were associated with a lower dental age in children. The children of mothers with severe vitamin D deficiency [25(OH)D <25.0 nmol/L] during midpregnancy exhibited a higher dental age (β: 0.14; 95% CI: 0.03, 0.24) and higher developmental stages of the mandibular first premolar (β: 0.32; 95% CI: 0.04, 0.60) compared with the children of mothers with optimal values of 25(OH)D (≥75.0 nmol/L). Children with vitamin D deficiency [25(OH)D 25.0–49.9 nmol/L] at birth exhibited a higher dental age (β: 0.11; 95% CI: 0.01, 0.20), higher developmental stages of the mandibular second premolar (β: 0.27; 95% CI: 0.02, 0.51), and higher developmental stages of the mandibular second molar (β: 0.24; 95% CI: 0.00, 0.48) compared with children with sufficient-to-optimal values of 25(OH)D (≥50.0 nmol/L) at birth.

**Conclusion:**

Higher maternal and neonatal 25(OH)D concentrations are associated with decelerated dental development in childhood. The lower the vitamin D level during midpregnancy or at birth, the higher the dental age of children, and the higher the developmental stages of the mandibular teeth.

## Introduction

Dental development is influenced by various biomarkers which inhibit or prohibit a cascade of signaling pathways (1–4). The earliest histologic sign of tooth formation is indicated by thickening of the oral epithelium on day 11 of gestation (5). The permanent dentition begins formation during midpregnancy, around the 20^th^ week of gestation, however matrix secretion will only start at birth (5). Micronutrient deficiency at these two essential time points can directly influence matrix secretion in the hard tissues of the teeth and consequently disturb the continuation of dental formation and mineralization (6–8). Calcium and phosphorus are the most important minerals that form the hydroxyapatite crystals of enamel (9); hence, hypocalcemia and hypophosphatemia can lead to impaired mineralization and delayed eruption of teeth (10).

Vitamin D plays an important role in calcium and phosphorus homeostasis by increasing their absorption in the intestines (11). Studies performed with rats demonstrate the implication of vitamin D in cell proliferation and differentiation during tooth development (12, 13). Therefore, a relation between the blood concentration of 25-hydroxyvitamin D [25(OH)D] and the initiation of tooth formation and mineralization in young individuals can be speculated (14).

Vitamin D (representing D2 and/or D3) in circulation is bound to the vitamin D binding protein which transports it to the liver, where vitamin D is converted to 25(OH)D and is the major circulating form used to determine vitamin D status (15). Diet and sunlight are the main sources for our body to absorb vitamin D (11). As a result of inadequate exposure to ultraviolet B radiation of sunlight, pregnant women in the Netherlands are a target population at high risk of vitamin D deficiency (16). In experimental studies, vitamin D deficiency decreased enamel and dentine mineralization, whereas excess vitamin D led to irreversible changes in the calcification of teeth (17, 18). Hence, a balanced concentration of vitamin D during odontogenesis is important to avoid disturbance of enamel or dentin maturation. Since vitamin D influences the mineralization of dental tissues, a possible relation between the concentration of vitamin D in early life and the timing of dental development in childhood can be hypothesized.

Therefore, in a population-based prospective cohort study involving 3,770 mothers and their children in the Netherlands, we investigated the associations of maternal and neonatal 25(OH)D concentrations with the timing of dental development of 10-y-old children.

## Methods

### Study design

This study was embedded in the Generation R Study, a population-based prospective cohort study from fetal life onward in Rotterdam, the Netherlands (19). All children were born between April 2002 and January 2006. Enrollment in the study was aimed at early pregnancy but was allowed until the birth of the child. The study protocol was approved by the Medical Ethical Committee of the Erasmus Medical Centre, Rotterdam, the Netherlands (MEC-2012–165) (20). Written consent was obtained from all participating mothers.

### Study population

Second-trimester 25(OH)D concentrations were measured in 7,934 mothers. For the present study, we excluded pregnancies that led to twin births (*N* = 77), children who did not attend follow-up visits at the age-10 assessment (*N* = 3,077), and children without an available dental panoramic radiograph (DPR) or bad image (*N* = 1,010). Thus, the cohort for analysis comprised 3,770 subjects with available information on maternal and fetal 25(OH)D concentrations and child dental development (**Supplementary Figure 1**).

### Maternal and neonatal 25(OH)D blood concentrations

Maternal venous blood samples were collected in the second trimester (median 20.4 weeks of gestation; range: 18.5–23.2 wk) whereas umbilical cord blood samples were collected immediately after delivery (median 40.1 weeks of gestation; range 35.9–42.3 wk). Measurements of 25(OH)D concentrations were conducted at the Eyles Laboratory of the Queensland Brain Institute, University of Queensland, Australia, in 2014. Total 25(OH)D concentrations were calculated as the sum of 25-hydroxyvitamin D2[25(OH)D2] and 25-hydroxyvitamin D3[25(OH)D3] measured in plasma, as previously described (21). Samples were quantified with the use of isotope dilution LC-tandem MS. The linearity of 25(OH)D concentrations was assessed with the use of matrix-matched calibration standards, with R^2^ values of >0.99 across the calibration range (10–125 nmol/L). Inter-assay inaccuracy and imprecision were assessed at four concentrations for 25(OH)D3 (48.3, 49.4, 76.4, and 139.2 nmol/L) and a single concentration (32.3 nmol/L) for 25(OH)D2 with the use of certified reference materials and were excellent at all concentrations tested. Inter-assay inaccuracy and imprecision were both <10% for 25(OH)D3 and <17% for 25(OH)D2, respectively. We categorized vitamin D status into quartiles by using cut-off concentrations according to previous recommendations (severely deficient: <25.0 nmol/L; deficient: 25.0–49.9 nmol/L; sufficient: 50.0–74.9 nmol/L; and optimal: ≥75.0 nmol/L) (22, 23).

### Dental development assessment

Dental development was defined using the Demirjian method based on the available DPRs of 10-y-old children. According to the Demirjian method, the calculation of dental age is derived from the developmental stages of the teeth present in the lower left quadrant (24). The lower jaw was advantageously chosen instead of the upper jaw because of the higher bone compactness and thus ease of precisely assessing the developmental stages of teeth from X-ray images. One experienced examiner (BD) determined the eight stages of development (1 to 8) for each of the seven permanent teeth located in the lower left quadrant (excluding the third molar) (24). If a permanent tooth in the left mandible was congenitally missing, the stage of development was assessed from the corresponding tooth in the right mandible. The obtained stages of development were weighted for boys and girls using the Dutch dental age standard (25). Finally, the summed dental maturity score was converted into dental age using the standard tables for each sex.

### Covariates

Information on sex and gestational age at birth was available from medical records and hospital registries. The date of blood sampling was categorized into summer, fall, winter, and spring, based on the European seasons. We obtained information on maternal age at intake, ethnicity, educational level, alcohol use, and folic acid and vitamin supplementation during pregnancy using questionnaires (20). Dietary calcium and phosphorus intake during pregnancy was measured at enrollment using a validated semiquantitative food-frequency questionnaire (SFFQ) (26). Ethnicity and educational level were defined according to the classification of Statistics Netherlands (27). For this study we categorized ethnicity into the following groups: European, Cape Verdean, Dutch Antillean, Moroccan, Surinamese, Turkish, and Other. Maternal prepregnancy height and weight were self-reported and prepregnancy BMI was calculated (kg/m^2^).

Measurements of the 25(OH)D concentration of children were assessed at a median age of 6 y (95% range, 5.6–7.9 y). Blood samples were drawn by antecubital venipuncture and stored at −80°C until analysis at the Endocrine Laboratory of the VU University Medical Center, Amsterdam, as previously described (28). Serum 25(OH)D was measured with the use of isotope dilution online solid phase extraction LC-tandem MS. Bone mineral density (BMD) of the head was ascertained at the age-6 assessment using an iDXA scanner (GE Healthcare). At the age-10 assessment, child height was determined in the standing position to the nearest millimeter without shoes using a Harpendenstadiometer (Holtain Limited). Weight was measured using a mechanical personal scale (SECA). We calculated child BMI using weight and height measured at the age-10 assessment. One experienced examiner ascertained hypodontia from the DPRs. Children were classified with hypodontia if no sign of tooth formation or calcification was shown in the DPR. Most children with hypodontia were missing 1 to 2 teeth and were not excluded from the study population as the Demirjian method takes missing teeth into account. Covariates were included in the regression models based on previous literature or a change of >10% in effect estimates (29, 30).

### Statistical analyses

We calculated the intra-class correlation (ICC) to test the agreement between two independent examiners who assessed stages of development (1 to 8) for each of the seven left mandibular teeth in a random subsample of 100 DPRs from the study population. The ICC for the scored teeth ranged between 0.65–0.80 which is considered to be in substantial agreement according to the conventional criteria (31). Mandibular central incisors were not taken into account due to the absence of variation in the stage of tooth development fitting with the age of the children.

To study the associations of maternal and neonatal vitamin D with the dental age of the children, we built three multivariate linear regression models. In Model 1, we adjusted for maternal-related confounders including season of blood sampling and season at birth, maternal age at intake, maternal BMI at intake, maternal ethnicity, education, alcohol consumption, folic acid intake, vitamin supplementation, calcium and phosphorus intake during pregnancy, and child-related confounders included age, hypodontia, BMI, and height. To control for confounding bias of vitamin D concentration of children during the 10-y timespan between exposure and outcome measurements, we included the 25(OH)D concentration of children measured at the age-6 assessment in Model 2. To control for any possible influence of jaw compactness in the studied association, we added the head BMD of children measured at the age-6 assessment as a potential confounder in Model 3. Maternal and neonatal 25(OH)D concentrations were analyzed continuously per SD increase in order to present countable values and to easily compare the effect estimates obtained from the multivariate linear regression models. Furthermore, we applied categorization of maternal and neonatal 25(OH)D concentrations based on the recommended clinical cut-offs, to explore the studied associations in more detail. We built three multivariate generalized linear models adjusted for confounders in the same consecutive steps. For neonatal 25(OH)D concentration, groups of optimal and sufficient 25(OH)D were joined to equalize the size of samples, and sufficient-to-optimal concentration of 25(OH)D was used as the reference group in the statistical analysis.

One fully adjusted ordinal regression model was built to study the association of maternal and neonatal 25(OH)D concentrations with developmental stages of the mandibular second molar, second premolar, first premolar, and canine. The model was adjusted for maternal-related confounders, child-related confounders, vitamin D status, and head BMD. The mandibular first molar, lateral incisor, and central incisor were in the final stage of calcification at the age of 10 y, hence they were left out of the statistical analysis.

We performed a nonresponse analysis by comparing the general characteristics between children with and without measurements of dental development, using the t-test, chi-square test, and Mann-Whitney test. The nonlinear associations were assessed by adding quadratic terms of maternal and neonatal 25(OH)D concentrations to the regression models. We tested for interactions between sex and 25(OH)D concentrations (maternal and neonatal) in relation to the dental age of children. Similarly, the interaction term between ethnicity and 25(OH)D concentrations (maternal and neonatal) in relation to the dental age of children was also tested. Since no interaction term result was statistically significant, we did not perform a stratification analysis. The Markov Chain Monte Carlo imputation method was used to reduce potential bias associated with missing data (0.01–22.8%) (32). Five imputed datasets were generated from which the pooled effect estimates are presented in this study (β; 95% CI). All results were considered statistically significant for a *P* value ≤0.05. All statistical analyses in this study were performed using Statistical Package for Social Sciences version 21.0 (SPSS Inc.).

## Results

### Subject characteristics

The general characteristics of the study population are presented in **Table 1** and **Table 2**. The median value (95% range) of 25(OH)D concentration in the second trimester was 52.50 (7.9–121.9) nmol/L, whereas the concentration of 25(OH)D at birth was lower with a median (95% range) of 30.7 (5.4–81.9) nmol/L. Among the 10-y-old children of the mothers included in the study, 5.2% had hypodontia. The mean dental age of children was 10.34 y (SD =  ±0.83 y). The development of mandibular canine, first premolar, second premolar, and second molar presented a median value of 6 mineralization stages; whereas the mandibular central incisor, mandibular lateral incisor, and first molar presented a median value of 8 mineralization stages.

**TABLE 1:** Characteristics of mothers included in the study (*N* = 3,770)^1^

Maternal characteristics	Value	Missing (N, %)
Maternal age, y	30.75 ± 4.8	
Gestational age at blood sampling, wk	20.36 (18.5–23.2)	231 (6.1)
Ethnicity, N, %		138 (3.7)
Dutch	2,097 (55.6)	
Cape Verdean	145 (3.8)	
Dutch Antillean	76 (2.0)	
Moroccan	189 (5.0)	
Turkish	248 (6.6)	
Surinamese	272 (7.2)	
Other	605 (16.0)	
BMI, kg/m^2^	23.66 (18.8–35.6)	23 (0.01)
Education, N, %		192 (5.1)
No education	7 (0.2)	
Primary	271 (7.2)	
Secondary	1,491 (39.5)	
Higher	1,809 (48.0)	
Alcohol consumption during pregnancy, N, %		436 (11.6)
Never	1,423 (37.7)	
Until pregnancy was known	489 (13.0)	
Continued	1,422 (37.7)	
Folic acid supplement, N, %		858 (22.8)
No use	614 (16.3)	
Start when pregnancy was known	930 (24.7)	
Periconceptional start	1,368 (36.3)	
Vitamin supplement use, N, %		581 (15.4)
Yes	1,069 (28.4)	
No	2,120 (56.2)	
Calcium intake, mg	1,117.27 (375.4–2093.6)	802 (21.3)
Phosphorus intake, mg	1,482.48 (655.5–2414.5)	802 (21.3)
Season when maternal blood sample was taken, N, %		231 (6.1)
spring	1,035 (27.5)	
summer	711 (18.9)	
autumn	874 (23.2)	
winter	919 (24.4)	
25(OH)D concentration (nmol/L) in midpregnancy	52.60 (7.9–121.9)	232 (6.2)
Severely deficient (<25.0), N, %	718 (19.0)	
Deficient (25.0–49.9), N, %	938 (24.9)	
Sufficient (50.0–74.9), N, %	905 (24.0)	
Optimal (≥75.0), N, %	977 (25.9)	

1 Values are percentages for categorical variables, means ± SD for continuous variables with a normal distribution, or medians (95% range) for continuous variables with a skewed distribution from the original data, 25(OH)D, 25-hydroxyvitamin D.

**TABLE 2:** Characteristics of children included in the study (*N* = 3,770)^1^

Child characteristics	Value	Missing (N, %)
Season of birth, N, %		1,311 (34.8)
spring	731 (19.4)	
summer	703 (18.6)	
autumn	511 (13.6)	
winter	514 (13.6)	
25(OH)D concentration (nmol/L) at birth	30.7 (5.4–81.9)	1,311 (34.8)
Severely deficient (<25.0), N, %	975 (25.9)	
Deficient (25.0–49.9), N, %	932 (24.7)	
Sufficient (50.0–74.9), N, %	444 (11.8)	
Optimal (≥75.0), N, %	108 (2.9)	
Gender (N, %)		–
Boys	1,873 (49.7)	
Girls	1,897 (50.3)	
Chronological age, y	9.81 ± 0.35	–
Ethnicity, N, %		60 (1.6)
Dutch	2,221 (58.9)	
Cape Verdean	112 (3.0)	
Dutch Antillean	107 (2.8)	
Moroccan	207 (5.5)	
Turkish	242 (6.4)	
Surinamese	263 (7.0)	
Other	558 (14.8)	
Weight, kg	34.00 (25.2–54.1)	–
Height, cm	141.72 ± 6.75	–
BMI, kg/m^2^	16.99 (14.0–24.7)	–
25(OH)D, nmol/L	66.20 (21.1–136.9)	1536 (40.7)
Bone mineral density of head, g/cm^2^	1.35 (1.1–1.6)	333 (8.8)
Dental age, y	10.34 ± 0.83	–
Stage of development for the central incisor	8 (8–8)	–
Stage of development for the lateral incisor	8 (7–8)	–
Stage of development for the canine	6 (5–8)	–
Stage of development for the first premolar	6 (5–7)	–
Stage of development for the second premolar	6 (4–7)	–
Stage of development for the first molar	8 (7–8)	–
Stage of development for the second molar	6 (4–7)	–
Hypodontia, N, %	197 (5.2)	–
Dental anomalies of position, N, %	102 (2.7)	

1 Values are percentages for categorical variables, means ± SD for continuous variables with a normal distribution, or medians (95% range) for continuous variables with a skewed distribution from the original data. 25(OH)D, 25-hydroxyvitamin D.

Results from the nonresponse analyses are given in **Supplementary Table 1**. Maternal and neonatal 25(OH)D concentrations did not differ significantly between participants and nonparticipants.

### The association between maternal 25(OH)D concentration and dental age of children

#### Analyzed continuously per SD increase

The increase of maternal 25(OH)D concentration was statistically significantly associated with decelerated dental age (**Table 3**). The effect estimates barely changed from Model 1 (β = −0.04; 95% CI: −0.07, −0.01) to Model 3 (β = −0.04; 95% CI: −0.08, −0.01).

**TABLE 3:** The association between total vitamin D concentration in midpregnancy and dental age (*N* = 3,538)^1^

	Model 1			Model 2			Model 3		
Total vitamin D	β	95% CI	*P* value	β	95% CI	*P* value	β	95% CI	*P* value
**Total vitamin D** nmol/L (continuous-SDS increase)	−0.04	−0.07, −0.01	0.016*	−0.04	−0.07, −0.00	0.029*	−0.04	−0.08, −0.01	0.017*
**Total vitamin D** nmol/L (clinical cut-offs)									
Optimal (≥75.0 nmol/L; ref)	–	–	–	–	–	–	–	–	–
Sufficient (50.0–74.9 nmol/L)	0.02	−0.05, 0.10	0.566	0.02	−0.06, 0.10	0.625	0.04	−0.04, 0.12	0.274
Deficient (25.0–49.9 nmol/L)	0.03	−0.05, 0.10	0.528	0.02	−0.06, 0.10	0.632	0.04	−0.04, 0.12	0.343
Severely deficient (<25.0 nmol/L)	0.14	0.04, 0.24	0.007*	0.13	0.03, 0.23	0.013*	0.14	0.03, 0.24	0.012*

1 Model 1: adjusted for season at gestational blood sampling, maternal age, BMI at intake, ethnicity, education, alcohol consumption, folic acid use, vitamin supplementation, calcium intake, phosphorus intake, age of child, hypodontia, and child BMI and height; Model 2: additionally adjusted for child vitamin D status; Model 3: additionally adjusted for head BMD of child. *Significant *P* value. BMD, bone mineral density; ref, reference; SDS, standard deviation score.

#### Analyzed in quartile categories by applying the recommended clinical cut-offs

Children of mothers with a severely low 25(OH)D concentration during midpregnancy had a higher dental age compared with the children of mothers with an optimal vitamin D 25(OH)D concentration, as shown in Model 1 (β = 0.14; 95% CI: 0.04, 0.23). The effect estimate changed slightly when either the 25(OH)D concentration of children at the age of 6 y was considered in Model 2 (β = 0.13; 95% CI: 0.03, 0.23), or when head BMD of children at the age of 6 y was included in Model 3 (β = 0.14; 95% CI: 0.03, 0.24).

### The association between neonatal 25(OH)D concentration and dental age of children

#### Analyzed continuously per SD increase

The increase of neonatal 25(OH)D concentration was statistically significantly associated with decelerated dental age (**Table 4**). The effect estimates barely changed from Model 1 (β = −0.05; 95% CI: −0.09, −0.02) to Model 3 (β = −0.06; 95% CI: −0.10, −0.02).

**TABLE 4:** The association between total vitamin D concentration at birth and dental age (*N* = 2,459)^1^

	Model 1			Model 2			Model 3		
Total vitamin D	β	95% CI	*P* value	β	95% CI	*P* value	β	95% CI	*P* value
**Total vitamin D** nmol/L (continuous-SDS increase)	−0.05	−0.09, −0.02	0.006*	−0.05	−0.09, −0.01	0.028*	−0.06	−0.10, −0.02	0.008*
**Total vitamin D** nmol/L (tertiles)									
Sufficient-optimal (≥50.0 nmol/L; ref)	–	–	–	–	–	–	–	–	–
Deficient (25.0–49.9 nmol/L)	0.11	0.03, 0.20	0.012*	0.10	0.01, 0.19	0.029*	0.11	0.01, 0.20	0.028*
Severely deficient (<25.0 nmol/L)	0.10	−0.00, 0.20	0.055	0.08	−0.03, 0.18	0.149	0.08	−0.03, 0.19	0.134

1 Model 1: adjusted for season of birth, maternal age, BMI at intake, ethnicity, education, alcohol consumption, folic acid use, vitamin supplementation, calcium intake, phosphorus intake, age of child, hypodontia, and child BMI and height; Model 2: additionally adjusted for child vitamin D concentration; Model 3: additionally adjusted for head BMD of child. *Significant *P* value. BMD, bone mineral density; ref, reference; SDS, standard deviation score.

#### Analyzed in three categories according to the recommended clinical cut-offs

Children with a deficiency of 25(OH)D concentration at birth had a higher dental age than children with sufficient-to-optimal values of 25(OH)D concentration at birth, as shown in Model 1 (β = 0.11; 95% CI: 0.03, 0.20). The effect estimate changed slightly when either the 25(OH)D concentration of children at the age of 6 y was added in Model 2 (β = 0.10; 95% CI: 0.01, 0.19), or when head BMD of children at the age of 6 y was considered in Model 3 (β = 0.11; 95% CI: 0.01, 0.20).

### The association of maternal 25(OH)D concentration with the development of mandibular teeth of children (**Figure 1**)

**FIGURE 1:**
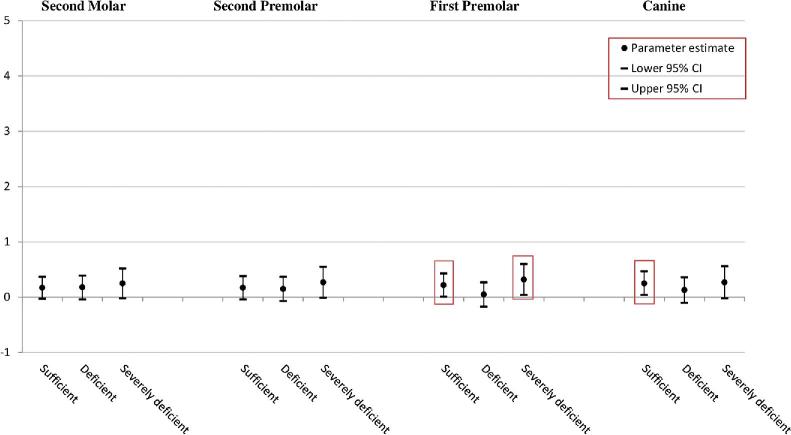
The association between vitamin D concentration in midpregnancy (clinical cut-offs) and developmental stages of the mandibular teeth. The ordinal regression model was fully adjusted for season at blood sampling, maternal age, BMI at intake, ethnicity, education, alcohol consumption, folic acid use, vitamin supplementation, age of child, hypodontia, child BMI and height, calcium and phosphorus intake, child vitamin D concentration, and head BMD; Y-axis represents developmental stages of the mandibular teeth. All the statistically significant data points are presented inside the squares. Optimal (≥75.0 nmol/L; reference); sufficient (50.0–74.9 nmol/L); deficient (25.0–49.9 nmol/L); severely deficient (<25.0 nmol/L). BMD, bone mineral density.

#### The canine

In comparison with an optimal maternal 25(OH)D concentration, children of mothers with a sufficient concentration of 25(OH)D during midpregnancy had accelerated developmental stages of the mandibular canine (β = 0.25; 95% CI: 0.04, 0.47).

#### The first premolar

In comparison with an optimal maternal 25(OH)D concentration, children of mothers with sufficient 25(OH)D concentration during midpregnancy had accelerated developmental stages of the mandibular first premolar (β = 0.22; 95% CI: 0.01, 0.43). Moreover, children of mothers with a severe deficiency of 25(OH)D concentration exhibited accelerated developmental stages of the mandibular first premolar (β = 0.32; 95% CI: 0.04, 0.60).

The ordinal regression analysis revealed no statistically significant difference among the categories of maternal 25(OH)D concentration during midpregnancy and developmental stages of the mandibular second premolar and second molar.

### The association of neonatal 25(OH)D concentration with the development of the mandibular teeth of children (**Figure 2**)

**FIGURE 2:**
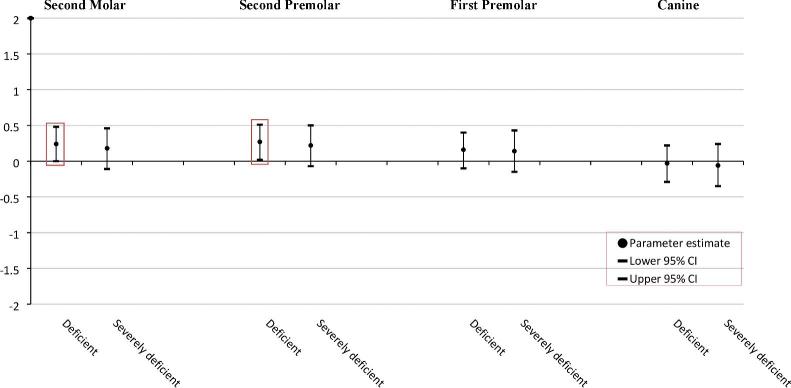
The association between vitamin D concentration at birth (clinical cut-offs) and developmental stages of the mandibular teeth. The ordinal regression model was fully adjusted for season of birth, maternal age, BMI at intake, ethnicity, education, alcohol consumption, folic acid use, vitamin supplementation, age of child, hypodontia, child BMI and height, calcium and phosphorus intake, child vitamin D concentration, and head BMD; Y-axis represents developmental stages of the mandibular teeth. All the statistically significant data points are presented inside the squares. Sufficient-optimal (≥50.0 nmol/L; reference); deficient (25.0–49.9 nmol/L); severely deficient (<25.0 nmol/L).

#### The second premolar

In comparison with an optimal-to-sufficient neonatal 25(OH)D concentration, children with a deficiency of 25(OH)D concentration at birth showed accelerated developmental stages of the mandibular second premolar (β = 0.27; 95% CI: 0.02, 0.51).

#### The second molar

In comparison with an optimal-to-sufficient neonatal 25(OH)D concentration, children with a deficiency of 25(OH)D concentration at birth had accelerated developmental stages of the mandibular second molar (β = 0.24; 95% CI: 0.00, 0.48).

The ordinal regression analysis revealed no statistically significant difference among the categories of neonatal 25(OH)D concentration and developmental stages of the mandibular canine and first premolar.

## Discussion

Results from this large population-based prospective cohort study suggest that maternal and neonatal 25(OH)D concentrations are inversely associated with the timing of childhood dental development.

### Interpretation of the main findings

Our hypothesis on the associations of maternal and neonatal 25(OH)D concentrations with the timing of dental development was based on reports that emphasize the role of vitamin D in early cellular proliferation and differentiation, and in tooth mineralization at the beginning of odontogenesis (33, 34). Compared with the children of mothers with an optimal 25(OH)D concentration during midpregnancy, the children of mothers with a sufficient 25(OH)D concentration during midpregnancy had accelerated developmental stages of the mandibular canine and first premolar; children of mothers with a severe deficiency of 25(OH)D concentration during midpregnancy showed accelerated developmental stages of the mandibular first premolar. Furthermore, compared to children with a sufficient-to-optimal 25(OH)D concentration at birth, children deficient in 25(OH)D concentration at birth showed accelerated developmental stages of the mandibular second premolar and second molar. Maternal 25(OH)D concentration was measured around the 20^th^ week of gestation when initial formation of the permanent dentition starts, whereas neonatal 25(OH)D concentration was measured at birth when the mineralization process begins (5). Referring to the timeline of human tooth development, the mandibular canine and mandibular first premolar, form, erupt, and completely develop earlier than the mandibular second premolar and mandibular second molar (**Supplementary Table 2**) (5, 35). Thus, the detection of the influence of maternal and neonatal 25(OH)D concentrations on the developmental stages of the respective teeth is mostly supported.

Referring to the importance of adequate prenatal vitamin D concentration in decreasing the risk of hypomineralization and decay of dental tissues in children, positive correlations of maternal and neonatal 25(OH) concentrations with the acceleration of dental development in children could be expected from our investigation (36–39). Although the role of an optimal vitamin D concentration on healthy development and mineralization of dental tissues is predominately underlined in the literature, recent studies attenuate the importance of vitamin D by showing weak or no correlation in their findings (40, 41). For example, van der Tas et al. showed no associations of fetal, neonatal, and child vitamin D status with the presence of enamel hypomineralization (14). Furthermore, the findings of Elamin and Liversidge showed no effect of malnutrition on timing of dental development in children, which was explained by the biological stability of teeth to nutrient deficiency (42). Accordingly, the importance of vitamin D deficiency on the timing of dental development appears questionable. We showed no significant association of severe deficiency of vitamin D at birth with accelerated dental age, although a tendency towards an existing association could be assumed considering the positive value of the effect estimate. However, severe deficiency of maternal 25(OH)D concentration and deficiency of neonatal 25(OH)D concentration were associated with accelerated dental development. A possible explanation for this finding could be the relation of prenatal malnutrition in early critical periods of development with rapid catch-up growth in childhood via biochemical pathways (43). As a consequence, accelerated development in childhood is predicted, including the timing of dental maturation, as a component of the general growth of an individual. Another explanation of our findings could be repression of tooth development enabled by vitamin D at the molecular level. Directly or indirectly, vitamin D controls >200 genes including genes responsible for cellular proliferation and differentiation. Previous studies have reported a link between vitamin D and the expression of paired box 9 gene (PAX9) and Msh homeobox 1 (MSX1) (44, 45). PAX9 and MSX1 are expressed during tooth formation in both the dental epithelium and mesenchyme of the human tooth germ from the cup stage (46). Moreover, genetic mutations of PAX9 and MSX1 cause oligodontia, known as the developmental failure of ≥6 teeth (47, 48). Ramenzoni et al. showed that vitamin D2 can increase the expression of the PAX9 gene (44). Furthermore, Lézot et al. showed a relation between vitamin D3 and overexpression of MSX1 activity (45). Although developmental disturbances of teeth can occur due to the overexpression of PAX9 and MSX1, vitamin D is not the only factor thought to be involved in the underlying molecular mechanism. Parathyroid hormone (PTH) may also have direct and indirect effects on tooth formation (49). In cases of vitamin D deficiency, PTH secretion is increased until 25(OH)D reaches a sufficient concentration (23). Hypoparathyroidism has been associated with the delayed eruption of teeth (50). On the other hand, hyperparathyroidism is associated with an increased alkaline phosphatase concentration, which plays an important role in dental maturation by increasing the velocity of the calcification process (9, 49). Further investigations performing detailed analysis at the molecular level with specific information on the expression activity of PAX9 and MSX1, also involving 25(OH)D concentration and PTH secretion, could shed light on our findings.

### Strength and limitations

To our knowledge, this is the largest multiethnic population-based prospective cohort study focused on the associations of maternal and neonatal 25(OH)D concentrations with the timing of dental development in children. For mothers with available 25(OH)D concentrations, we had a limited loss to follow-up; therefore, we do not expect biased results from selective follow-up. We used the 25(OH)D concentration, which is the best and most widely used indicator of vitamin D status. Moreover, we analyzed vitamin D concentrations continuously and applied the clinical cut-offs (22, 23). In line with recommendations from the Endocrine Society and based on previous results from the Generation R Study and other cohort studies, we created 4 vitamin D groups, including severely deficient (<25.0 nmol/L), deficient (25.0–49.9 nmol/L), sufficient (50.0–74.9 nmol/L), and optimal (≥75.0 nmol/L) (51, 52). Although the categories are useful for comparisons and to avoid nonlinearity of the studied associations, sample size and statistical power decreases. In the time span of 10 y between measurements of the exposure and the outcome, a number of other factors should be considered. Important nutritional factors in children linked to being breastfed or formula-fed, age of weaning, and concentrations of calcium and vitamin D in children, especially prior to the age of 6 y, were not taken into consideration and should be counted as a limitation of the study. The lack of detailed information on vitamin D supplementation is also counted as a limitation of this study. Vitamin D supplementation is linked to high awareness of vitamin D deficiency (11, 23). The Health Council of the Netherlands suggests that all pregnant women should take 10 μg of vitamin D daily (53); however, this recommendation has not been implemented in the national guidelines for general practitioners and midwives (54). On the other hand, taking multivitamin supplementation (5–10 μg daily) during pregnancy decreases the risk of vitamin D deficiency (23, 55). Hence in our analyses, we considered vitamin supplementation estimated from the questionnaire filled in during pregnancy. Lack of information on additional factors that may influence vitamin D status, such as other nutritional factors, maternal lifestyle, sunlight exposure, and vitamin D content of diets are also counted as limitations (54, 55). We included calcium and phosphorus in our regression models because previous studies have suggested that these micronutrients influence dental development (10). As the intake of calcium and phosphorus was estimated from an FFQ, the precision of the concentrations may not have been achieved. Dark skin is protective against the intense sunlight at the equator but at other latitudes, with low sunlight intensity, individuals with dark skin are vulnerable to vitamin D deficiency (55). Women of a non-Dutch ethnic background are at particularly high risk of vitamin D deficiency (54). Ethnicity showed no interacting effect on the studied associations, and different influence of early-life vitamin D status on dental development in childhood among specific ethnicities could therefore not be reported. We estimated dental development from the developmental stages of the left mandibular teeth and calculated the dental age of each child. Extending the assessment of dental development by adding more measurements such as ascertaining the number of erupted teeth should be considered for future investigations. A longitudinal approach to assess dental development in children would also be necessary.

In conclusion, the lower the maternal and neonatal vitamin D concentration, the higher the dental age of the children and the higher developmental stages of the mandibular canine, first premolar, second premolar, and second molar. These findings highlight the importance of balanced concentrations of 25(OH)D in the critical time points of tooth formation during pregnancy.

## Supplementary Material

Supplement File

## Notes

We declare no external funding for the preparation of this manuscript.

Author disclosures: BD, KM, TV, HWT, VWVJ, EBW, and EMO, no conflicts of interest.

Supplemental Tables 1 and 2 and Supplemental Figure 1 are available from the “Supplementary data” link in the online posting of the article and from the same link in the online table of contents at https://academic.oup.com/cdn/.

Abbreviations used:

BMD: bone mass density
DPR: dental panoramic radiograph
ICC: intra-class-correlation
MSX1: Msh homeobox 1
PAX9: paired box 9 gene
PTH: parathyroid hormone
SFFQ: semiquantitative food-frequency questionnaire
25(OH)D: 25-hydroxyvitamin D
25(OH)D2: 25-hydroxyvitamin D2
25(OH)D3: 25-hydroxyvitamin D3
