# Supplementary material for: The Associations of Maternal and Neonatal Vitamin D with Dental Development in Childhood
Source: Curr Dev Nutr. 2019 Mar 7;3(4):nzy100. doi: 10.1093/cdn/nzy100 (PMC6435444; doi:10.1093/cdn/nzy100)
Supplement: Supplement File [file nzy100_supplement_file.docx]

**Online Supporting Material**

**Supplementary figure 1.** Flow chart of the study participants

Available information on 25(OH) D concentration in mid-pregnancy and at birth

**N = 7,934**

N = 5,562

**N = 77** excluded due to twin births

Singleton life born children

**N = 7,857**

**N = 3,077** excluded children who did not attend follow up visits at the age of 10 years

Children participating at age-10 assessment follow up measurements

**N = 4,780**
N = 3,877

**N = 1,010** excluded children without an available DPR or bad image

Final population for analysis

with available measurements on maternal and neonatal 25(OH) D concentration and also child dental development **N=3,770**

**Online Supporting Material**

| **Supplementary table 1a.** Characteristics of non-participants in the follow-up measurements of dental development included in the study (N=1010) | | | |
| --- | --- | --- | --- |
| ***Maternal characteristics*** | **Participation**  (N=3770) | **No-participation** (N=1010) | **p-value** |
| Maternal age (years) | 30.75 (4.83) | 30.63 (5.12) | 0.477 |
| Gestational age at blood sampling (weeks) | 20.36 (18.5-23.2) | 20.64 (18.7-35.8) | *<0.001* |
| Missing (N; %) | 231 (6.1) | 187 (18.5) |  |
| Ethnicity (N, %) |  |  | 0.772 |
| Dutch | 2097 (55.6) | 556 (55.5) |  |
| Cape Verdean | 145 (3.8) | 38 (3.8) |  |
| Dutch Antillean | 76 (2.0) | 26 (2.6) |  |
| Moroccan | 189 (5.0) | 44 (4.4) |  |
| Turkish | 248 (6.6) | 76 (7.5) |  |
| Surinamese | 272 (7.2) | 73 (7.2) |  |
| Other | 605 (16.0) | 154 (15.2) |  |
| Missing (N; %) | 138 (3.7) | 43 (4.3) |  |
| Body mass index (kg/m^2^) | 23.66 (18.8-35.6) | 23.99 (18.8-35.8) | 0.078 |
| Missing (N, %) | 23 (0.01) | 2 (0.002) |  |
| Education (N; %) |  |  | 0.386 |
| No education | 7 (0.002) | 4 (0.004) |  |
| Primary | 271 (7.2) | 71 (7.0) |  |
| Secondary | 1491 (39.5) | 426 (42.2) |  |
| Higher | 1809 (48.0) | 452 (44.8) |  |
| Missing | 192 (5.1) | 57 (5.6) |  |
| Alcohol consumption during pregnancy |  |  | 0.309 |
| Never | 1423 (37.7) | 393 (38.9) |  |
| Until pregnancy was known | 489 (13.0) | 115 (11.4) |  |
| Continued | 1422 (37.7) | 399 (39.5) |  |
| Missing (N, %) | 436 (11.6) | 103 (10.2) |  |
| Folic acid supplement (N, %) |  |  | *0.018* |
| No use | 614 (16.3) | 195 (19.3) |  |
| Start when pregnancy was known | 930 (24.7) | 254 (25.1) |  |
| Periconceptional start | 1368 (36.3) | 326 (32.3) |  |
| Missing | 858 (22.8) | 235 (23.3) |  |
| Vitamin supplement use |  |  | 0.119 |
| Yes | 1069 (28.4) | 590 (58.4) |  |
| No | 2120 (56.2) | 269 (26.6) |  |
| Missing (N, %) | 581 (15.4) | 151 (15.0) |  |
| Calcium intake (mg) | 1117.27 (375.4-2093.6) | 1034.76 (352.3-2042.7) | *<0.001* |
| Missing (N, %) | 802 (20.9) | 173 (17.1) |  |
| Phosphorus intake (mg) | 1482.48 (655.5-2414.5) | 1429.59 (606.3-2393.5) | *0.029* |
| Missing (N, %) | 802 (21.3) | 173 (17.1) |  |
| Season when maternal blood sample was taken |  |  | *<0.001* |
| Spring | 1035 (27.5) | 241 (23.9) |  |
| Summer | 711 (18.9) | 260 (25.7) |  |
| **Online Supporting Material** (continuing) | | | |
| Autumn | 874 (23.2) | 229 (22.7) |  |
| Winter | 919 (24.4) | 93 (9.2) |  |
| Missing (N, %) | 231 (6.1) | 187 (18.5) |  |
| 25(OH)D concentration (nmol/L) in mid-pregnancy | 52.60 (7.9-121.9) | 51.10 (8.2-125.4) | 0.734 |
| Severely deficient (<25.0 nmol/L) | 718 (19.0) | 172 (17.0) |  |
| Deficient (25.0-49.9 nmol/L) | 938 (24.9) | 216 (21.4) |  |
| Sufficient (50.0-74.9 nmol/L) | 905 (24.0) | 196 (19.4) |  |
| Optimal (≥75.0 nmol/L) | 977 (25.9) | 239 (23.7) |  |
| Missing (N, %) | 232 (6.2) | 187 (18.5) |  |
| Values are percentages for categorical variables, means (SD) for continuous variables with a normal distribution, or medians (95% range) for continuous variables with a skewed distribution; Differences were tested using independent t-test for continuous variables, chi-squared test for categorical variables and Mann-Whitney Non-Parametric test for variables with a skewed distribution, using participation group as the reference; Significant *p-values* are presented in italic font | | | |

**Online Supporting Material**

| **Supplementary table 1b.** Characteristics of non-participants in the follow-up measurements of dental development included in the study (N=1010) | | | |
| --- | --- | --- | --- |
| ***Child characteristics*** | **Participation**  (N=3770) | **No-participation** (N=1010) | **p-value** |
| Season of birth |  |  | *<0.001* |
| Spring | 731 (19.4) | 85 (8.4) |  |
| Summer | 703 (18.6) | 155 (15.3) |  |
| Autumn | 511 (13.6) | 214 (21.2) |  |
| Winter | 514 (13.6) | 240 (23.8) |  |
| Missing (N, %) | 1311 (34.8) | 316 (31.3) |  |
| 25(OH)D concentration (nmol/L) at birth | 30.7 (5.4-81.9) | 28.40 (4.7-87.1) | 0.137 |
| Severely deficient (<25.0 nmol/L) | 975 (25.9) | 304 (30.1) |  |
| Deficient (25.0-49.9 nmol/L) | 932 (24.7) | 242 (24.0) |  |
| Sufficient (50.0-74.9 nmol/L) | 444 (11.8) | 114 (11.3) |  |
| Optimal (≥75.0 nmol/L) | 108 (2.9) | 34 (3.4) |  |
| Missing (N, %) | 1311 (34.8) | 316 (31.3) |  |
| Gender (N, %) |  |  | 0.183 |
| Boys | 1873 (49.7) | 485 (48.0) |  |
| Girls | 1897 (50.3) | 525 (52.0) |  |
| Chronological age (years) | 9.81 (0.35) | 9.78 (0.47) | *0.023* |
| Ethnicity (N, %) |  |  | 0.578 |
| Dutch | 2221 (58.9) | 587 (58.1) |  |
| Cape Verdean | 112 (3.0) | 27 (2.7) |  |
| Dutch Antillean | 107 (2.8) | 37 (3.7) |  |
| Moroccan | 207 (5.5) | 46 (4.6) |  |
| Turkish | 242 (6.4) | 75 (7.4) |  |
| Surinamese | 263 (7.0) | 68 (6.7) |  |
| Other | 558 (14.8) | 148 (14.7) |  |
| Missing (N, %) | 60 (1.6) | 22 (2.2) |  |
| Weight (kg) | 34.00 (25.2-54.1) | 33.6 (25.0-53.9) | 0.080 |
| Missing (N, %) | - | 136 (13.5) |  |
| Height (cm) | 141.72 (6.75) | 141.26 (6.46) | 0.069 |
| Missing (N,%) | - | 137 (13.6) |  |
| Body mass index (kg/m2) | 16.99 (14.0-24.7) | 16.98 (13.9-25.3) | 0.994 |
| Missing (N, %) | - | 137 (13.6) |  |
| 25(OH)D (nmol/L) | 66.20 (21.1-136.9) | 60.00 (15.0-116.5) | *<0.001* |
| Missing (N, %) | 1536 (40.7) | 458 (45.3) |  |
| Bone mineral density of head (g/cm^2^) | 1.35 (1.1-1.6) | 1.34 (1.1-1.6) | 0.165 |
| Missing (N; %) | 333 (8.8) | 128 (12.7) |  |
| Values are percentages for categorical variables, means (SD) for continuous variables with a normal distribution, or medians (95% range) for continuous variables with a skewed distribution; Differences were tested using independent t-test for continuous variables, chi-squared test for categorical variables and Mann-Whitney Non-Parametric test for variables with a skewed distribution, using participation group as the reference; Significant *p-values* are presented in italic font | | | |

**Online Supporting Material**

| **Supplementary Table 2.** Timeline of tooth development | | | |
| --- | --- | --- | --- |
|  | Initial formation | Eruption | Complete Calcification |
| Mandibular canine | 0.3-0.4 years | 9-10 years | 12-14 years |
| Mandibular first premolar | 1.5-1.75 years | 10-12 years | 12-13 years |
| Mandibular second premolar | 2.25-2.5 years | 11-12 years | 13-14 years |
| Mandibular second molar | 2.5-3 years | 11-13 years | 14-15 years |
